# Prognostic and therapeutic implications of BRCA1 and BRCA2 mutations in ovarian cancer

**DOI:** 10.3389/fonc.2026.1899836

**Published:** 2026-09-08

**Authors:** Weronika Pawul, Paulina Opoka, Agata Dusza, Bernard Blajerski, Julia Orzelska, Natalia Gierulska, Krzysztof Kułak, Rafał Tarkowski

**Affiliations:** 1 Student’s Scientific Association at the I Chair and Department of Gynaecological Oncology and Gynaecology, Medical University of Lublin, Lublin, Poland; 2 I Chair and Department of Gynaecological Oncology and Gynaecology, Medical University of Lublin, Lublin, Poland

**Keywords:** BRCA 1/2 mutation, BRCA1/2, genetic testing, HRD (homologous recombination deficiency), ovarian cancer, PARPi, PARPi resistance, treatment

## Abstract

**Introduction:**

Ovarian cancer remains one of the leading causes of cancer-related mortality among women because of its asymptomatic early course, delayed diagnosis, and frequent presentation at advanced stages. Germline and somatic mutations in the BRCA1 and BRCA2 genes significantly increase the risk of ovarian cancer by impairing homologous recombination DNA repair, resulting in genomic instability. Beyond their role in carcinogenesis, BRCA mutations have become important prognostic and predictive biomarkers, influencing treatment response and clinical outcomes. This review summarizes current evidence regarding the impact of BRCA1/2 status on prognosis and therapeutic strategies in ovarian cancer.

**Methods:**

A literature review was conducted using the PubMed and Scopus databases. English-language articles published between 2016 and 2026 were identified using combinations of the keywords *ovarian cancer*, *BRCA1*, *BRCA2*, *PARP inhibitors*, *homologous recombination deficiency*, and *treatment*. Original studies, systematic reviews, meta-analyses, and relevant clinical trials were included.

**Results:**

The available evidence demonstrates that patients carrying BRCA1 or BRCA2 mutations generally experience improved progression-free survival and, in many studies, prolonged overall survival compared with non-carriers. This survival advantage is largely attributed to increased sensitivity to platinum-based chemotherapy and the efficacy of PARP inhibitors, which exploit synthetic lethality in homologous recombination-deficient tumors. The literature also emphasizes the importance of universal BRCA testing to optimize treatment selection, identify candidates for maintenance PARP inhibitor therapy, and facilitate genetic counseling. In addition, emerging mechanisms of resistance to PARP inhibitors remain a significant therapeutic challenge.

**Conclusion:**

BRCA1/2 mutation status is a key biomarker in the management of ovarian cancer, supporting personalized treatment strategies and improving patient outcomes. Further research is needed to identify novel predictive biomarkers, overcome therapeutic resistance, and develop more effective targeted therapies.

## 1 Introduction

Ovarian cancer is the sixth most common cancer in women and one of the most dangerous. It has a high mortality rate, making it the fifth most common cause of death among women (1, 2). This is primarily due to the difficulty of diagnosis in the early stages of the cancer. The clinical picture of ovarian cancer is non-specific, and the symptoms reported by patients, such as abdominal pain, back pain, bloating and urinary tract symptoms, are non-specific even in advanced stages of the cancer and can be attributed to other diseases with similar symptoms (1, 3). Problems also arise at the laboratory diagnosis stage, as there are no suitable biomarkers with high sensitivity and specificity. In clinical practice, the CA-125 biomarker concentration and the ROMA algorithm are used, which, although undoubtedly helpful in monitoring the disease, still do not guarantee early and reliable diagnosis. All this makes ovarian cancer a difficult disease to diagnose and one that is often diagnosed at an advanced stage (FIGO III and IV), when it is essential to initiate appropriate treatment quickly (4, 5).

Ovarian cancer most commonly affects women aged 55–70 (2). Risk factors include early menarche, late menopause, obesity and infertility, but the most important factor is a positive family history. The risk of developing the disease in the general population is approximately 1-2%, but it increases significantly if the patient has a BRCA1 (39%-44%) or BRCA2 (11%-17%) mutation. The BRCA mutation is inherited in an autosomal dominant manner, therefore genotyping of relatives of patients diagnosed with ovarian cancer allows for earlier risk assessment and implementation of appropriate prevention measures, which is also extremely important due to the younger age of onset in patients with the BRCA mutation (6, 7). The co-occurrence of mutations and ovarian cancer, as well as breast cancer, is called hereditary breast and ovarian cancer syndrome (HBOC). Studies show that patients who develop HBOC respond better to chemotherapy than patients without mutations (8). Cancer cells in tumours associated with the BRCA mutation are more sensitive to platinum-based chemotherapy. Unfortunately, resistance to platinum drugs is increasingly observed. In such cases, it is possible to include PARP inhibitors, which also show promising treatment results (9).

The five-year survival rate varies, depending on the stage of the cancer at the time of diagnosis. The highest rate - 92.1% - is achieved if ovarian cancer is diagnosed at FIGO stage I, while at FIGO stage III/IV it drops to 25% (10). We can also maintain longer survival times in patients with the mutation (11). It also varies, depending on the histological subtype of thecancer, among which we can distinguish: epithelial cancers such as serous, mucinous and endometrial, which constitute the vast majority (90%), and non-epithelial cancers. Serous ovarian cancer, which is also the most common ovarian cancer, is most often associated with the BRCA mutation (2, 12). Endometrial and clear cell subtypes may co-occur with the BRCA mutation, but there is no evidence of a strong correlation. In contrast, mucinous ovarian cancer is virtually absent in patients with the mutation (7). In terms of five-year survival, the most aggressive subtype is serous carcinoma, with a survival rate of 43%, while other subtypes have a better prognosis: endometrial (82%), mucinous (71%) and clear cell (66%) (13).

All of this makes BRCA1/2 mutations significant in the prevention and early diagnosis of ovarian cancer in genetically predisposed patients. The aim of this publication is to summarise the available knowledge on BRCA mutations and to highlight their association with prognosis and treatment outcomes in patients with ovarian cancer.

## 2 Materials and methods

The literature search was conducted based on PRISMA guidelines. The following databases were searched: PubMed and Scopus. The final literature search was completed on May 15, 2026. The following keywords were used: “ovarian cancer”, “BRCA1/2”, and “treatment.” In the Pubmed database, using the phrase “ovarian cancer” AND (“brca1” OR “brca2”) resulted in 4, 280 records, while refining the query to the form “ovarian cancer” AND (“brca1” OR “brca2”) AND “treatment” allowed for the identification of 1, 241 items. Similar search strategies were applied to the Scopus database, resulting in 10, 255 and 3, 228 findings, respectively. 

Inclusion criteria: studies published between 2016 and 2026; publications in English; topics directly related to ovarian cancer with a concomitant BRCA gene mutation. Exclusion criteria: studies published in a language other than English; publications where access was limited to the abstract only; studies that relate to a small study group and are not reflected in current guidelines (e.g. case reports). While the authors acknowledge that excluding small study groups and case reports carries a potential risk of omitting rare clinical phenomena, this decision was deliberately made to align the analysis with high-level evidence and current clinical guidelines. The focus of this review was restricted to established therapeutic trends and statistically robust data, ensuring that the synthesized conclusions remain generalizable and directly applicable to standard clinical practice.

**Figure 1:**
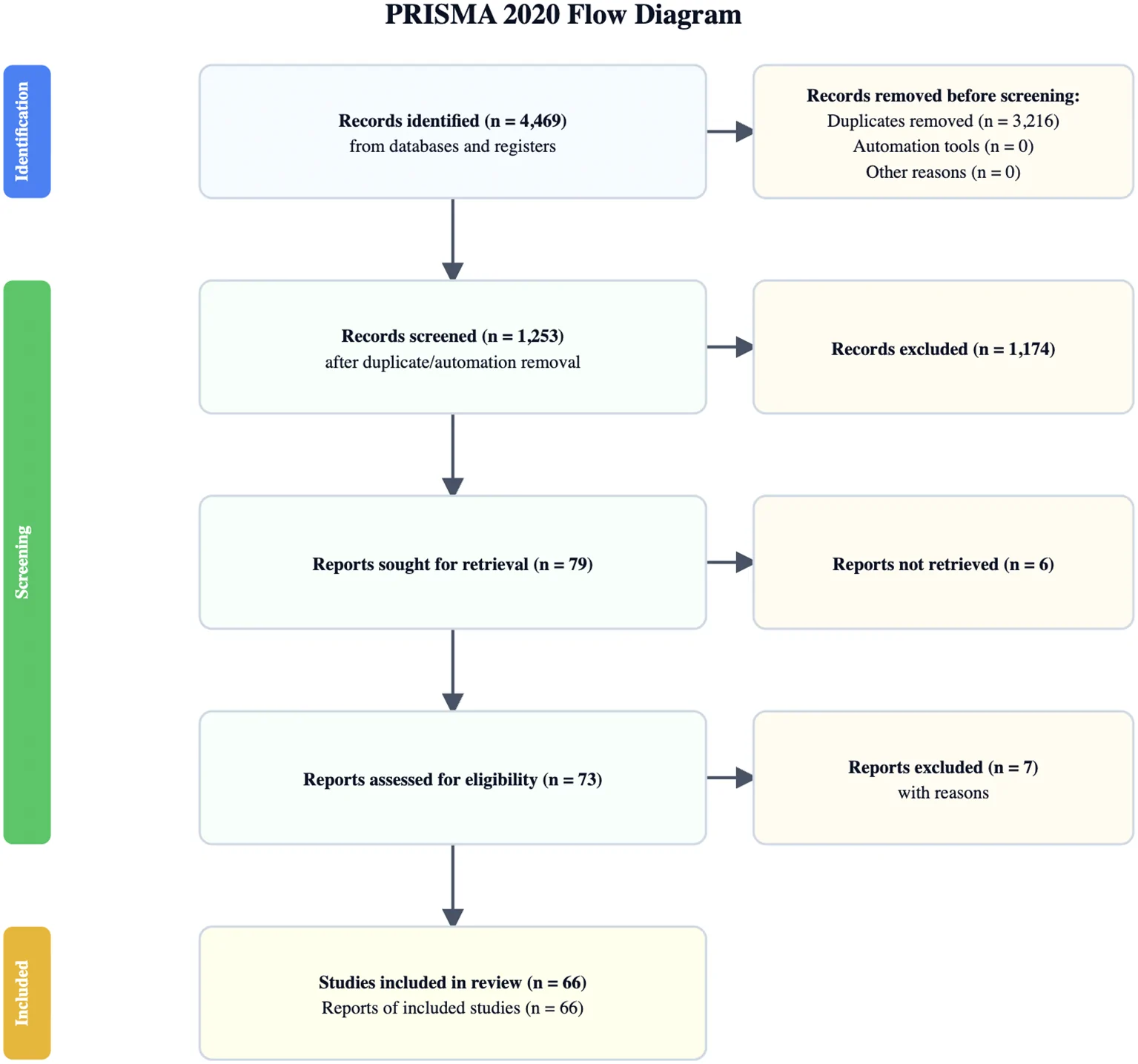
PRISMA 2020 flow diagram illustrating the study selection process. Of the 1, 253 screened records, 1, 174 were excluded based on title and abstract screening (reasons: 820 lacked direct relevance to BRCA1/2-mutated ovarian cancer, 210 focused on non-ovarian malignancies, and 144 lacked BRCA-specific focus). Of the 73 full-text articles assessed for eligibility, 7 were excluded with specific reasons: 3 due to insufficient clinical outcome data, 2 due to duplicate patient cohorts, and 2 due to non-English language. A total of 66 studies met all inclusion criteria and were included in the review.

The process of identifying and selecting materials was based on a systematic approach. Following an initial search and the removal of duplicates, the remaining articles were assessed for compliance with the established inclusion criteria. The substantive relevance of the available literature was verified through a two-stage manual screening process conducted independently by the authors. In the first stage, titles and abstracts were screened to eliminate studies unrelated to clinical outcomes or therapeutic interventions. In the second stage, full-text articles were thoroughly evaluated to ensure they provided specific data regarding treatment efficacy, clinical management, or patient outcomes strictly in the context of ovarian cancer with concomitant BRCA1/2 mutations. During this screening process, 1, 174 articles were explicitly excluded after title and abstract evaluation due to non-compliance with the specific inclusion criteria (e.g., studies focused on other gynecological malignancies or breast cancer rather than ovarian cancer, or lack of focus on BRCA1/2 mutations). Of the 73 articles identified for full-text review, 66 were ultimately used for the final analysis and the writing of this publication. The study selection process and the flow of literature search are summarized in the PRISMA flow diagram (**Figure 1**).

To ensure methodological rigor, the quality assessment and risk of bias evaluation of the included studies were integrated into the literature selection process using strict inclusion and exclusion criteria. Preliminary reports, case reports, and studies with small sample sizes (low statistical power), which could introduce a high risk of bias, were deliberately excluded from the analysis. The selection of literature and verification of data reliability were conducted independently by the authors, and any doubts regarding the methodological consistency of the analyzed papers were resolved through consensus. This approach ensured that the analysis was restricted exclusively to peer-reviewed, full-text publications with high scientific credibility.

## 3 Results

### 3.1 Characteristics of BRCA genes

The BRCA1 and BRCA2 genes participate in DNA repair processes and colocalize in nuclear foci with the RAD51 protein, a key component of homologous recombination within a likely widespread DNA repair pathway (14). The BRCA1 gene is located on chromosome 17q21, whereas the BRCA2 gene is located on chromosome 13q12 (14).

DNA, as the carrier of genetic information, is continuously subjected to damage caused by endogenous and exogenous factors. Its integrity is maintained by mechanisms responsible for the repair of single-strand DNA breaks (ssDNA), double-strand DNA breaks (dsDNA), and mismatch repair (MMR). Depending on the type of damage, base excision repair (BER) and nucleotide excision repair (NER) pathways are activated. BRCA1 and BRCA2 are tumor suppressor genes involved in the repair of double-strand DNA breaks via homologous recombination repair (HRR), playing a crucial role in maintaining genomic stability, as evidenced by the impairment of this process in BRCA1-mutated cell lines. Both proteins also participate in the activation and maintenance of the G2/M cell cycle checkpoint, preventing the segregation of damaged chromosomes into daughter cells. Beyond canonical double-strand break repair, BRCA1 is involved in the response to replication stress by promoting homologous recombination at sites of single-ended DNA lesions arising from stalled replication forks and by limiting tandem repeat accumulation. These functions are mediated through the formation of distinct BRCA1 complexes with various DNA repair factors, the detailed mechanisms of which remain under investigation (9, 15).

Additionally, BRCA1 and BRCA2 regulate centromere dynamics, chromosome segregation, and cytokinesis, ensuring proper genomic control throughout the cell cycle. They also participate in the repair of DNA interstrand crosslinks, defects of which underlie Fanconi anemia. Both proteins have been shown to prevent the accumulation of RNA: DNA hybrids in the form of R-loops, further protecting genomic integrity. Although BRCA1 and BRCA2 perform distinct functions in homologous recombination and DNA replication, it remains unclear to what extent defects in replication fork protection and suppression of single-stranded DNA gaps contribute to tumorigenesis, particularly given that BRCA1/2 heterozygous cells retain normal levels of homologous recombination but exhibit defects in these additional protective mechanisms (9, 15).

In families with a high hereditary cancer risk, protein truncating variants (PTVs) located in exon 11 of the BRCA1 gene are associated with a reduced risk of breast cancer and an increased risk of ovarian cancer compared with PTVs occurring in other regions of the gene. For this reason, exon 11 has been designated as the ovarian cancer cluster region (OCCR). Similarly, PTVs in exon 11 of the BRCA2 gene are associated with an increased risk of ovarian cancer (BRCA2 OCCR) and a decreased risk of breast cancer compared with PTVs located outside this exon (16).

Carriers of mutations in exon 11, and thus within the OCCR, may exhibit a distinct phenotype compared with individuals carrying other BRCA1 mutations. This is related to the formation of a characteristic truncated BRCA1 protein. From an epidemiological perspective, it is estimated that approximately 50–67% of BRCA1 and BRCA2 mutations identified in patients with ovarian cancer occur within OCCR regions (17).

The relative risk of cancer development varies depending on the location of the mutation within the OCCR. However, the reported risk ratios resulting from differences in mutation position generally do not exceed approximately two or are lower, which makes their application in individual risk assessment and clinical decision-making unjustified (15).

In addition to germline and somatic mutations in homologous recombination (HR) pathway genes, homologous recombination deficiency (HRD) may also result from epigenetic mechanisms leading to silencing of HR gene expression, including BRCA1 and BRCA2. BRCA dysfunction may also occur in the absence of direct mutations or epigenetic alterations through indirect mechanisms involving disruption of BRCA protein interactions with other components of the DNA repair machinery (18).

The clinical significance of germline BRCA mutations has been well characterized. Carriers of germline mutations have an increased risk of developing cancers such as breast, ovarian, prostate, and pancreatic cancer, and these malignancies often occur at a younger age compared with the general population. Individuals with a detected germline mutation should undergo intensive oncological surveillance along with the implementation of appropriate cancer prevention strategies. Family members of individuals with a mutation should also be offered genetic testing and clinical monitoring. Despite extensive knowledge regarding germline mutations, the role of somatic mutations in these genes remains under intensive investigation. It is not yet fully clear whether the biological consequences of somatic BRCA1 and BRCA2 mutations are equivalent to those observed in hereditary mutations. Analogous to germline mutations, the presence of somatic BRCA mutations has been associated with prolonged progression-free survival (PFS) compared with wild-type tumors, although no significant correlation with overall survival has been demonstrated. The presence of both germline and somatic mutations was a strong predictor of primary sensitivity to platinum-based chemotherapy (P = 0.0002) and was associated with improved overall survival (P = 0.0006), although the analysis was not restricted exclusively to BRCA1 and BRCA2 genes (18).

### 3.2 Epidemiology and histology of BRCA mutations

**Figure 2:**
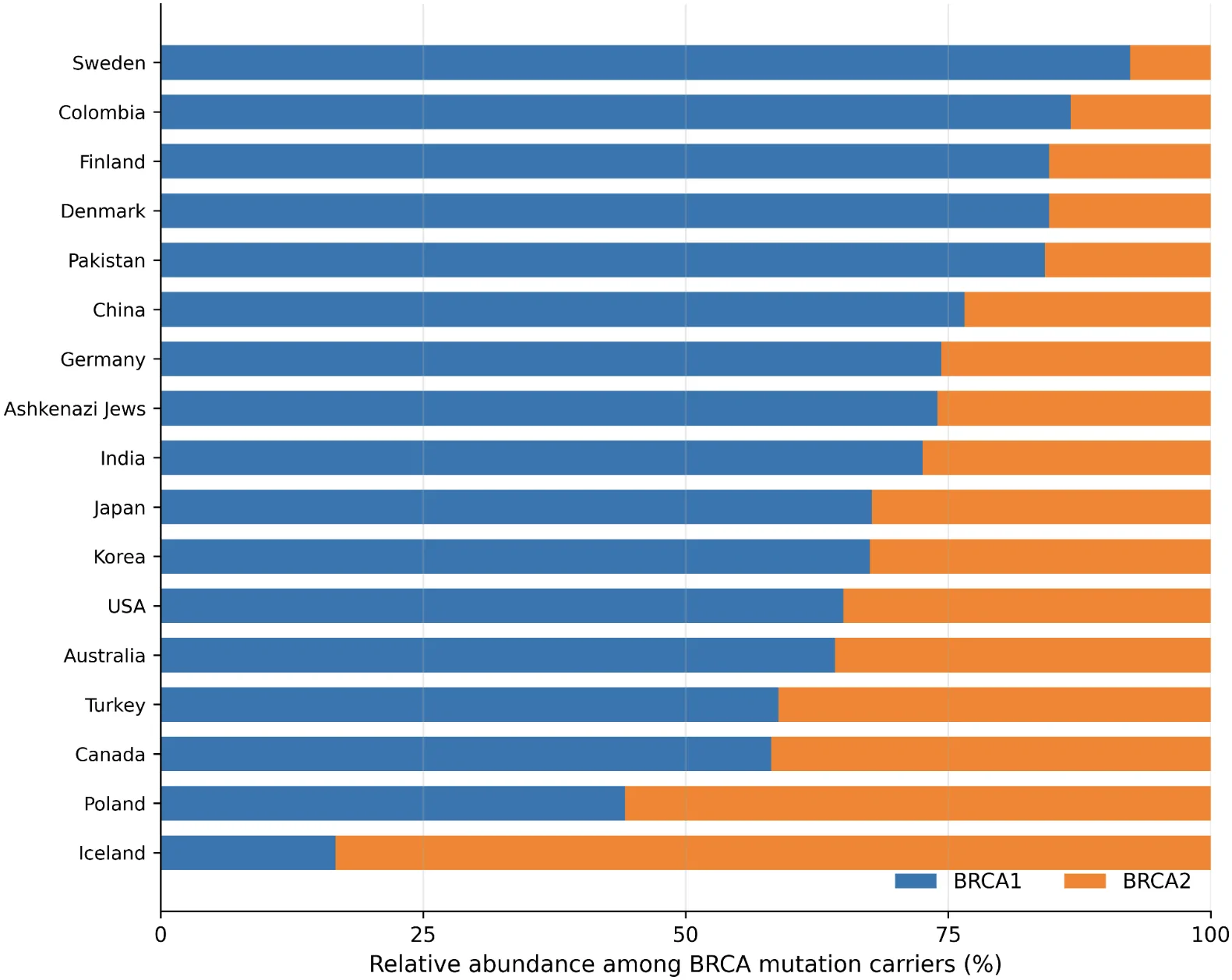
Relative abundance of BRCA1 and BRCA2 mutations among ovarian cancer patients across different countries and populations.

Literature data indicates that over 20% of all ovarian cancer cases are associated with changes within the genome, of which approximately 70% are germline mutations in the BRCA1 and BRCA2 genes, responsible for normal DNA damage repair mechanisms (12). The significance of these mutations is confirmed by their high penetrance in ovarian cancer, which is approximately 40% for the BRCA1 gene and 20% for the BRCA2 gene (19). The presence of defects in the DNA repair pathway leads to genomic instability, which significantly increases the risk of malignant transformation of ovarian epithelial cells. The prevalence of BRCA1/2 germline mutations in the ovarian cancer population varies considerably and is strongly influenced by ethnicity and geographic region. Epidemiological data indicates that the percentage of BRCA mutation carriers ranges from 5% to 30%, with the highest prevalence observed in the Ashkenazi Jewish population. In countries such as the United States, Canada, Australia, and Japan, the prevalence of BRCA germline mutations remains at an average level of approximately 15%, while in selected European countries, including Finland, Sweden, Denmark, and Iceland, relatively lower values are observed (7). The relative abundance and geographic distribution of these mutations across the discussed populations are illustrated in **Figure 2**.

The ratio of BRCA1 to BRCA2 mutations also varies between populations. Most reports indicate that germline BRCA1 mutations are more common than BRCA2 mutations in ovarian cancer patients. However, exceptions to this rule have been noted, for example, in Iceland and Poland, where BRCA2 mutations were more frequently identified (7). The reasons for this phenomenon have not been clearly explained, but it is suspected that the presence and prevalence of founder mutations characteristic of specific ethnic groups may play a significant role. It is important to note that somatic BRCA1/2 mutations also play a part in oncogenesis.

Studies that analyzed the somatic BRCA1/2 mutation frequencies have been summarized in **Table 1**.

**Table 1:** Reported BRCA1/2 somatic mutations in major ovarian cancer genomic datasets.

Dataset/publication	Ovarian cancer cohort	Sequencing and variant definition	BRCA1 finding	BRCA2 finding	Combined finding and interpretation
TCGA Research Network, 2011 (20)	489 high-grade serous ovarian adenocarcinomas; coding exome sequencing in 316 tumors.	Matched tumor-normal exome sequencing with integrated copy-number, methylation and expression analyses. The main text reports somatic BRCA1/2 sequence mutations jointly.	3% combined with BRCA2	3% combined with BRCA1	Somatic mutations in BRCA1 or BRCA2 occurred in a further 3% of cases, in addition to germline BRCA1 (9%) and BRCA2 (8%) mutations. This 3% estimate primarily reflects coding sequence variants and should not be interpreted as the frequency of all structural or epigenetic BRCA defects.
AACR Project GENIE analysis (Ahsan et al., 2023) (21)	3, 304 high-grade serous ovarian carcinoma samples used as a comparator cohort in an AACR Project GENIE analysis.	Multi-institutional targeted clinical sequencing. Reported values are tumor-detected mutation frequencies; testing platforms and availability of matched normal samples were not uniform.	10%	7%	Gene-specific frequencies were reported, but a deduplicated combined BRCA1/2 frequency was not provided. These values should not be labelled as strictly somatic because occult germline variants may be present in tumor-only data; the source is a conference abstract.
MSK-IMPACT validation cohort (Vázquez-García et al., 2022) (22)	1, 298 patients with high-grade serous ovarian or fallopian-tube carcinoma; matched tumor-normal MSK-IMPACT sequencing.	Targeted tumor-normal panel sequencing. Stratified cases into BRCA1- and BRCA2-mutant subgroups for HLA loss-of-heterozygosity analysis.	116 BRCA1-mutant cases (approximately 8.9% of the full cohort).	81 BRCA2-mutant cases (approximately 6.2% of the full cohort).	The publication did not present these as somatic-only prevalence estimates; the figure-defined mutant groups may include both germline and somatic pathogenic alterations. The counts were reported as subgroup denominators rather than as the study’s primary prevalence endpoint.
PCAWG-containing WGS meta-cohort (Ewing et al., 2021) (23)	205 high-grade serous ovarian carcinomas: AOCS (n=80), TCGA (n=44) and Scottish HGSOC (n=81), analyzed using matched tumor-normal whole-genome sequencing.	Uniform whole-genome analysis of short variants, copy-number changes and large structural variants at BRCA1/2. The cohort incorporated datasets processed within PCAWG and additional WGS cases.	Heterozygous deletions spanning BRCA1 were observed in 16% of tumors.	Heterozygous deletions spanning BRCA2 were observed in 14% of tumors.	Structural variants independently affected 16% of patients beyond those with disruptive short BRCA1/2 variants; disruptive short variants in either gene were present in 24% (50/205). The 24% category included germline and somatic short variants, whereas the 16% estimate captures additional large structural disruption and is not equivalent to a conventional somatic point-mutation rate.

AOCS, Australian Ovarian Cancer Study; HGSOC, high-grade serous ovarian carcinoma; MSK-IMPACT, Memorial Sloan Kettering-Integrated Mutation Profiling of Actionable Cancer Targets; PCAWG, Pan-Cancer Analysis of Whole Genomes; TCGA, The Cancer Genome Atlas; WGS, whole-genome sequencing.

The estimates are not directly interchangeable. TCGA provides an explicitly somatic combined sequence-mutation estimate, whereas GENIE reports tumor-detected mutations, the MSK publication reports all BRCA-mutant subgroups rather than somatic-only prevalence, and the PCAWG-containing WGS analysis captures large structural alterations in addition to short variants. The rows should therefore be compared with the testing method and variant definition in mind.

Analysis of the relation between BRCA mutations and histological types of ovarian cancer clearly indicates the strongest association with high-grade serous carcinoma, which predominates among BRCA-related tumors. In the cited study, the percentage of BRCA mutations in high-grade serous carcinoma ranged from 16% to 28% and was significantly higher in Asian countries than in Western countries (11, 24). Other histotypes, such as endometrioid carcinoma and low-grade serous carcinoma, demonstrate a much weaker and more variable association with BRCA mutations, while mucinous and clear cell carcinomas only sporadically coexist with germline mutations in these genes (7, 12). This variation suggests a distinct mechanism of pathogenesis for each histological type of ovarian cancer and a limited role for DNA repair defects in some of them.

The practical conclusion is that the presence of BRCA mutations should be particularly considered in patients with high-grade serous carcinoma, both in the context of genetic diagnosis and the selection of targeted therapy, which further confirms the importance of precise histopathological classification in modern oncology.

### 3.3 Clinical impact and prognostic value

Pathogenic BRCA1/2 mutations in ovarian cancer have been associated with improved clinical outcomes; however, their independent prognostic value remains controversial and appears to depend on the biological and therapeutic context. Several studies suggest that BRCA mutations are associated with longer overall survival (OS) and a tendency toward improved progression-free survival (PFS), particularly in the era of targeted therapies. A cohort study including 50 patients, predominantly diagnosed with high-grade serous ovarian carcinoma (HGSC; 94%), evaluated the association between chemotherapy response score (CRS), BRCA1/2 mutation status, and clinical outcomes following neoadjuvant chemotherapy. Patients were classified as CRS1 (n=14; 28%), CRS2 (n=29; 58%), or CRS3 (n=7; 14%), and 96% underwent optimal or complete cytoreductive surgery. Pathogenic BRCA1/2 variants were most frequently identified in the CRS2 group (51.7%) and least frequently in CRS1 (7.1%). BRCA1/2 mutation carriers demonstrated a trend toward improved survival, with longer median PFS compared with non-carriers (42.43 vs. 22.24 months; P = 0.08) and significantly longer OS (64.32 vs. 56.63 months; P = 0.04). However, multivariate analysis did not confirm BRCA1/2 mutation status as an independent prognostic factor for either PFS or OS, while disease stage and optimal cytoreductive surgery remained the strongest predictors of survival. Moreover, CRS classification was not significantly associated with PFS or OS (25, 26).

The prognostic relevance of BRCA alterations appears to be highly tissue-dependent. A large study by Pourmansoni et al. (2026), based on The Cancer Genome Atlas (TCGA) cohorts of breast cancer (n=967) and ovarian cancer (n=571), evaluated the prognostic impact of TP53, BRCA1, and BRCA2 mutations. After adjustment for clinical variables, BRCA2 mutations were associated with significantly worse OS in breast cancer patients (HR = 3.91; 95% CI: 1.10–13.9; P = 0.034), whereas no significant prognostic effects were observed for BRCA1 or TP53 mutations. In contrast, BRCA2 mutations were associated with markedly improved OS in ovarian cancer patients (HR = 0.18; 95% CI: 0.04–0.82; P = 0.018). BRCA1 mutations showed a non-significant trend toward improved outcomes, while TP53 mutations were not associated with prognosis. These findings highlight that the clinical significance of BRCA mutations cannot be interpreted independently of tumor type and underlying biological mechanisms. The clinical importance of BRCA mutations becomes particularly evident in the context of PARP inhibitor therapy. The phase III SOLO-1 trial included 391 patients with newly diagnosed FIGO stage III–IV high-grade serous or endometrioid ovarian cancer harboring deleterious germline or somatic BRCA1/2 mutations. All patients achieved a complete or partial response following first-line platinum-based chemotherapy and were randomized to maintenance olaparib (n=260) or placebo (n=131) for up to 2 years. After approximately 7 years of follow-up, olaparib demonstrated sustained clinical benefit, with improved overall survival (HR = 0.55; 95% CI: 0.40–0.76; P = 0.0004). At 7 years, 67.0% of patients receiving olaparib were alive compared with 46.5% in the placebo group, and 45.3% remained alive without the need for subsequent anticancer therapy compared with 20.6% in the placebo group. Long-term safety remained favorable, with low incidence of myelodysplastic syndrome and acute myeloid leukemia, supporting the role of olaparib as an effective maintenance strategy in patients with BRCA1/2-mutated advanced ovarian cancer (27).

Long-term analyses of PARP inhibitor maintenance studies indicate that patients with BRCA mutations derive clinically meaningful improvements in survival, even when BRCA status alone does not demonstrate independent prognostic value in multivariate analyses. These findings suggest that the beneficial effect of BRCA mutations on survival is particularly pronounced when combined with modern maintenance treatment strategies rather than representing an isolated biological prognostic factor (22, 23). Pooled analyses demonstrated that BRCA1/2 mutations were associated with improved OS (HR = 0.75; 95% CI: 0.64–0.88; P<0.001) and PFS (HR = 0.80; 95% CI: 0.64–0.99; P = 0.039). BRCA1 mutations were associated with significantly improved OS, although not PFS, whereas BRCA2 mutations alone did not significantly influence OS or PFS. Furthermore, BRCA1/2 mutations were associated with a higher overall response rate to chemotherapy, suggesting that homologous recombination deficiency contributes to increased sensitivity to platinum-based treatment and PARP inhibition (28).

BRCA mutations also influence treatment response and may serve as predictive biomarkers for platinum chemotherapy and PARP inhibitor therapy. Patients with BRCA-mutated tumors generally demonstrate higher sensitivity to platinum-based chemotherapy and may achieve better responses to neoadjuvant treatment. However, the clinical value of response assessment tools such as the chemotherapy response score differs according to BRCA status. In patients with wild-type BRCA, CRS appears to have both prognostic and predictive value, whereas in BRCA-mutated patients its significance is reduced, indicating the need for additional predictive biomarkers in this subgroup (29).

It should also be emphasized that improvements in OS observed in PARP inhibitor trials persisted despite the frequent use of PARP inhibitors in subsequent treatment lines among control-group patients, which may have reduced the observed survival difference between treatment arms. This further supports the importance of initiating maintenance PARP inhibitor therapy early in the disease course of BRCA-mutated patients rather than delaying targeted treatment until relapse.

In conclusion, BRCA1/2 mutations represent clinically relevant biomarkers in ovarian cancer, influencing both prognosis and therapeutic response. Their favorable impact on survival appears to result from the combination of homologous recombination deficiency, increased sensitivity to platinum-based chemotherapy, and effectiveness of PARP inhibitor maintenance therapy. Although BRCA status alone does not consistently function as an independent prognostic factor, particularly after adjustment for surgical and clinical variables, its presence identifies a subgroup of patients who may achieve prolonged remission and improved survival with appropriate targeted treatment strategies (25, 27, 28).

### 3.4 BRCA mutations and therapy selection

Therapeutic methods for ovarian cancer have limited potential due to late diagnosis and their close dependence on the patient’s general condition (12, 30, 31). Surgical treatment is complemented by adjuvant chemotherapy. BRCA1/BRCA2 mutation status does not alter the sequence of treatment, and the choice between cytoreductive surgery and chemotherapy depends on the patient’s general condition. Additionally, a concomitant BRCA mutation increases tumor sensitivity to neoadjuvant chemotherapy and allows for a higher response rate. Therefore, chemotherapy is introduced as postoperative or neoadjuvant treatment, depending on the feasibility of primary cytoreduction (2, 11, 29).

Drugs used in the treatment of platinum-sensitive cancers include cisplatin, carboplatin, and oxaliplatin. Patients are typically treated with a combination of a platinum compound and paclitaxel. This therapeutic regimen is the basis of first-line therapy and the treatment of recurrent platinum-sensitive ovarian cancer (31). The mechanism of action of cisplatin, carboplatin, and, less commonly used, oxaliplatin, involves direct damage to tumor cells. This occurs by binding the drug, usually to guanine in the tumor’s DNA, leading to the formation of single damage and then to the formation of connections within one or both DNA strands. The damage results in disruption of DNA function, which leads to apoptosis of the cancer cell. BRCA mutations are a positive predictor of response to platinum-based therapy, and BRCAmut tumors are characterized by increased sensitivity to these drugs. This is due to the fact that in the case of co-occurrence of BRCA mutations, the function of the DNA repair pathway by homologous recombination (HR) is impaired, which leads to the accumulation of DNA damage (31, 32).

#### 3.4.1 PARP inhibitors

PARP inhibitor therapy for BRCA-mutated epithelial ovarian cancer is a relatively new treatment modality. The most commonly used drugs include olaparib, rucaparib, and niraparib. The mechanism of action of PARPi is the inhibition of PARP1 and PARP2 proteins, which, by excising BER bases, are critical enzymes responsible for the repair of single-stranded DNA breaks. Blocking their action leads to the accumulation of single-stranded damage in the tumor DNA, which results in double-strand breaks and, consequently, cell death. The particular effectiveness of this class of drugs in BRCAmut ovarian cancer is related to the dysfunction of the homologous recombination DNA repair pathway. HR deficiency contributes to the death of cancer cells whose DNA cannot be effectively repaired. The interaction of PARPi and the deficiency in homologous recombination, inherent to the nature of BRCAmut cancer, clearly contribute to improved efficacy of ovarian cancer treatment. The phenomenon of cell apoptosis resulting from the simultaneous loss of two mechanisms responsible for DNA repair is called synthetic lethality. In the absence of a BRCA mutation, the homologous recombination pathway functions normally, so the effectiveness of PARP inhibitors is significantly reduced. The treatment does not have to be lethal to cancer cells because DNA is effectively repaired via the HR pathway. Therefore, therapy using PARPi is particularly indicated in the treatment of ovarian cancers with BRCAmut, demonstrating significant efficacy (33, 34).

Olaparib is used for maintenance therapy in both newly diagnosed and advanced BRCAmut ovarian cancer. The absence of a detected mutation contributed to a smaller benefit, which was present only in the presence of abnormal homologous recombination of tumor cells, which is related to the mechanism described above (27, 34). Niraparib is a drug used for maintenance therapy in newly diagnosed advanced epithelial ovarian cancer. It is used for treatment regardless of BRCA1/2 mutation status (34, 35).

The results of the most important PARP inhibitor maintenance trials such as SOLO1, PAOLA-1 Study-19, NOVA, ARIEL3 have been summarised in **Table 2**.

**Table 2:** Comparative characteristics and efficacy outcomes of pivotal PARP inhibitor maintenance trials in ovarian cancer.

Trial and treatment	Patient population	BRCA/HRD status	Progression-free survival	Overall survival
SOLO1/GOG-3004; phase III; olaparib 300 mg twice daily for up to 2 years vs placebo; n=391 (24, 36)	Newly diagnosed FIGO stage III–IV high-grade serous or endometrioid ovarian, fallopian-tube, or primary peritoneal cancer Complete or partial response following first-line platinum-based chemotherapy.	A deleterious or suspected deleterious germline or somatic BRCA1/2 mutation was required.	median PFS 56.0 vs 13.8 months; HR 0.33 (95% CI 0.25–0.43). Five-year PFS rate: 48.3% vs 20.5%.	At 7 years: median OS not reached vs 75.2 months; HR 0.55 (95% CI 0.40–0.76). Seven-year OS rate: 67.0% vs 46.5%. The prespecified threshold for formal statistical significance was not crossed.
PAOLA-1/ENGOT-ov25; phase III; olaparib 300 mg twice daily plus bevacizumab vs placebo plus bevacizumab; n=806 (37, 38)	Newly diagnosed FIGO stage III–IV high-grade ovarian, fallopian-tube, or primary peritoneal cancer. Clinical response after first-line platinum–taxane chemotherapy plus bevacizumab; enrollment was independent of surgical outcome.	All-comer population. HRD-positive disease was defined as a tumor BRCA1/2 mutation and/or genomic-instability score ≥42. BRCA mutation was not required.	ITT: median PFS 22.1 vs 16.6 months; HR 0.59 (95% CI 0.49–0.72). HRD-positive: 37.2 vs 17.7 months; HR 0.33 (95% CI 0.25–0.45). HRD-positive/BRCA-wild-type: 28.1 vs 16.6 months; HR 0.43 (95% CI 0.28–0.66). No benefit in HRD-negative disease: 16.6 vs 16.2 months; HR 1.00.	ITT: median OS 56.5 vs 51.6 months; HR 0.92 (95% CI 0.76–1.12). HRD-positive: 75.2 vs 57.3 months; HR 0.62 (95% CI 0.45–0.85); f ive-year OS 65.5% vs 48.4%. HRD-negative: 36.8 vs 40.4 months; HR 1.19 (95% CI 0.88–1.63).
Study 19; randomized phase II; olaparib capsules 400 mg twice daily vs placebo; n=265 (39–41)	Platinum-sensitive recurrent high-grade serous ovarian, fallopian-tube, or primary peritoneal cancer. At least two previous platinum-based regimens; complete or partial response to the most recent regimen.	Not biomarker-selected; BRCA status was largely determined retrospectively. Both BRCA-mutated and BRCA-wild-type tumors were included.	Overall: median PFS 8.4 vs 4.8 months; HR 0.35 (95% CI 0.25–0.49). BRCAm: 11.2 vs 4.3 months; HR 0.18 (95% CI 0.10–0.31). BRCA wild-type: 7.4 vs 5.5 months; HR 0.54 (95% CI 0.34–0.85).	Overall: median OS 29.8 vs 27.8 months; HR 0.73 (95% CI 0.55–0.95); the prespecified significance threshold was not met. BRCAm: 34.9 vs 30.2 months; HR 0.62. BRCA wild-type: 24.5 vs 26.6 months; HR 0.83.
ENGOT-OV16/NOVA; phase III; niraparib 300 mg once daily vs placebo; n=553 (42, 43)	Platinum-sensitive recurrent ovarian cancer, predominantly of high-grade serous histology. At least two previous platinum regimens; complete or partial response to the most recent platinum treatment.	Two independently analyzed cohorts: germline BRCAm (n=203) and non-germline BRCAm (n=350); the latter was additionally evaluated according to HRD status.	gBRCAm: median PFS 21.0 vs 5.5 months; HR 0.27 (95% CI 0.17–0.41). Non-gBRCAm: 9.3 vs 3.9 months; HR 0.45 (95% CI 0.34–0.61). Non-gBRCAm/HRD-positive: 12.9 vs 3.8 months; HR 0.38 (95% CI 0.24–0.59).	Final OS in gBRCAm cohort: 40.9 vs 38.1 months; HR 0.85 (95% CI 0.61–1.20). Non-gBRCAm cohort: 31.0 vs 34.8 months; HR 1.06 (95% CI 0.81–1.37). No significant OS improvement was observed.
ARIEL3; phase III; rucaparib 600 mg twice daily vs placebo; n=564 (44, 45)	Platinum-sensitive recurrent high-grade serous or endometrioid ovarian, fallopian-tube, or primary peritoneal carcinoma. At least two previous platinum regimens; complete or partial response to the latest platinum therapy.	Three nested populations: tumor BRCAm; HRD, defined as BRCAm or BRCA-wild-type/LOH-high; and the overall ITT population.	Tumor BRCAm: median PFS 16.6 vs 5.4 months; HR 0.23. HRD: 13.6 vs 5.4 months; HR 0.32. ITT: 10.8 vs 5.4 months; HR 0.36 (95% CI 0.30–0.45).	Final OS: tumor BRCAm 45.9 vs 47.8 months, HR 0.83 (95% CI 0.58–1.19); HRD 40.5 vs 47.8 months, HR 1.01; ITT 36.0 vs 43.2 months, HR 1.00. No OS benefit was demonstrated; approximately 46% of placebo recipients receiving subsequent treatment were subsequently exposed to a PARP inhibitor.

BRCAm, BRCA1/2-mutated, CI-confidence interval; FIGO, International Federation of Gynecology and Obstetrics; gBRCAm, germline BRCA1/2-mutated; HR, hazard ratio; HRD, homologous recombination deficiency; ITT, intention-to-treat; LOH, loss of heterozygosity, NR-not reached; OS, overall survival; PARP, poly(ADP-ribose) polymerase; PFS, progression-free survival; PFS2, time to second progression or death; TFST, time to first subsequent therapy or death.

Outcomes are presented as experimental treatment versus control; survival medians are expressed in months. Cross-trial comparisons should be interpreted cautiously because SOLO1 and PAOLA-1 evaluated first-line maintenance, whereas Study 19, NOVA and ARIEL3 evaluated maintenance following platinum-sensitive recurrence. The trials also differed in biomarker selection, control treatment, PARP-inhibitor exposure duration, statistical power for OS and subsequent access to PARP inhibitors.

#### 3.4.2 Other therapies

In recent years, thanks to the enormous commitment of scientists and a significant number of clinical trials, many innovative methods of treating ovarian cancer have been developed. Immunotherapy has shown promising results and may have a significant impact in the future. It uses immune checkpoint inhibitors (ICI) that inhibit pathways that block T cell activity, such as PD-1, PD-L1, and CTLA-4, as well as drugs that act as antagonists to the glucocorticoid receptor (GR). A breakthrough drug is pembrolizumab, a monoclonal antibody that is a PD-1 inhibitor and an ICI. It is used in the treatment of recurrent ovarian cancer, platinum-resistant ovarian cancer and ovarian cancer with PD-L1 expression. Evidence of pembrolizumab’s efficacy was provided by the results of the phase III ENGOT-ov65/KEYNOTE-B96 trial. The addition of pembrolizumab to paclitaxel therapy, with or without bevacizumab, results in prolonged OS in the population of all treated patients. Results from the phase III ROSELLA trial demonstrated the efficacy of the drug - a GR antagonist. By blocking GRs, it modulates the ovarian tumour microenvironment (TME), thereby neutralising the TME’s potent immunosuppressive properties. Another drug with the same mechanism of action as pembrolizumab is nivolumab. Durvalumab acts in a similar manner, however, unlike the two previously mentioned drugs, it binds to PD-L1 expressed on tumor cells and blocks the inhibitory signal that suppresses the immune response. The use of the GR antagonist in combination with nab-paclitaxel (albumin-bound paclitaxel) improved PFS, including in patients who had received PARP inhibitor therapy, and demonstrated a provisional benefit in terms of OS in the ITT population (46).

Mirvetuximab is a modern anti-cancer antibody-drug conjugate (ADC) targeting folate receptor alpha (FRα). The mechanism of action of mirvetuximab involves the antibody binding to FRα on the surface of cancer cells and releasing a cytotoxic substance into the cells, leading to their death. High expression of FRα is more pronounced in cancer cells than in healthy tissues, which contributes to the drug’s efficacy. In the FORWARD I, FORWARD II, SORAYA and MIRASOL clinical trials, mirvetuximab demonstrated efficacy in platinum-resistant ovarian epithelial cancer and improved PFS in patients (47).

Immunotherapy has a low efficacy when used as monotherapy, and pembrolizumab remains the only drug approved for clinical use. Polytherapy brings better therapeutic results in many types of cancer, but contributes to increased toxicity of the therapy. Studies have shown that ICI can be combined with bevacizumab and PARP inhibitors, but efficacy depends on predictive biomarkers of response to ICI (35).

An alternative and complementary treatment modality for epithelial ovarian cancer is hyperthermic intraperitoneal chemotherapy (HIPEC), primarily used in patients with FIGO stage III disease. HIPEC involves the intraperitoneal administration of a heated chemotherapeutic agent, most commonly cisplatin. Elevated temperature exerts a direct cytotoxic effect on cancer cells, enhances the antitumor activity of the drug, and improves tissue penetration. Eligibility criteria for HIPEC include advanced epithelial ovarian cancer (FIGO stage III), prior neoadjuvant therapy, the possibility of achieving complete or partial cytoreduction, and, in the van Driel trial, residual disease of ≤1 cm. The results of randomized trials are inconsistent. In the study by Lim et al., the addition of HIPEC did not improve PFS or OS, whereas the OVHIPEC-1 trial demonstrated a significant improvement in both PFS and OS without a meaningful increase in the incidence of grade 3–4 adverse events. Current evidence suggests that the greatest therapeutic benefit may be achieved in patients with BRCAmut and platinum-sensitive tumors, particularly when HIPEC is combined with secondary cytoreductive surgery. However, further studies are needed to confirm these findings. Despite its potential advantages, HIPEC has not yet been widely adopted in routine clinical practice due to limited clinical experience, concerns regarding adverse effects, and inconsistent efficacy across certain subtypes of ovarian cancer (35, 48).

### 3.5 The importance of genetic testing

Genetic testing is important in the prevention, diagnosis and monitoring of ovarian cancer, especially in patients with a positive family history (2, 12). Next-generation sequencing (NGS) is primarily used to detect abnormalities in the genome. It allows for detection of both germinal and somatic BRCA mutations (18). Importantly, the co-occurrence of different mutations, may contribute to the progression of neoplasms in both women and men, most importantly pancreatic, breast and prostate cancers. Therefore, genetic assessment is important from the point of view of preventing other oncological diseases in all persons related to the patient (8). The absence of pathogenic mutations on genetic testing in individuals with a positive family history reduces the risk of ovarian cancer to that observed in the general population (6, 49).

Genetic testing in women at high risk of ovarian cancer enables the identification of individuals carrying BRCA mutations, allowing them to receive specialized surveillance and care. More frequent laboratory and imaging assessments may facilitate earlier detection of abnormalities. Moreover, studies have demonstrated that screening programs can be clinically valuable and effective in women with a genetic predisposition to ovarian cancer (9, 49).

Genetic counseling is offered to carriers of the mutated gene who plan to have children. The BRCA mutation can be detected during prenatal testing (6). For carriers of the mutated gene aged 35-45, the most frequently recommended surgical method for ovarian cancer prevention is the removal of the ovaries and fallopian tubes, i.e., bilateral salpingo-oophorectomy (BSO). BSO reduces the risk of ovarian, fallopian tube, and peritoneal cancers by up to 80%. However, it is important to note that this procedure is not recommended without valid indications, as it causes “surgical menopause” and contributes to a number of side effects (9, 30, 50). These can be mitigated with hormone replacement therapy. Studies indicate that oral contraceptives may help prevent ovarian cancer by inducing apoptosis of epithelial cells in these organs, but they do not provide a definitive answer. Other studies indicate that use of oral contraceptives may be a risk factor for ovarian cancer, which is why BSO remains the only preventive method with proven effectiveness (12, 51).

Ovarian cancer associated with a mutated BRCA gene demonstrates a better response to treatment with PARP inhibitors than cancers unrelated to the mutation. Therefore, genetic testing enables the selection of targeted treatment with higher efficacy. Studies have shown that treatment efficacy in patients without the mutation is comparable to that observed in the absence of treatment. By assessing the genetic background of the cancer, we can protect patients with a normal BRCA gene from the toxicity of treatment that will not produce the expected effects (25, 33).

### 3.6 Limitations of current knowledge and future challenges

Despite substantial advances in ovarian cancer therapy, several important limitations remain. Many clinical trials are non-randomized and involve relatively small patient cohorts, which may limit the objectivity and generalizability of their findings (49, 52). In addition, there is still a lack of effective screening methods for women at high risk of developing ovarian cancer (6). One of the most significant clinical challenges is the development of resistance to PARP inhibitors, which currently represent a cornerstone of treatment for BRCA-mutated ovarian cancer. Importantly, studies indicate that up to 40% of patients may derive limited or no clinical benefit from PARPi therapy (53).

#### 3.6.1 Progression and mechanisms of resistance to PARP inhibitors

There are many mechanisms that contribute to the progression of drug resistance, including mutations causing restoration of homologous recombination, increased drug efflux, or secondary stabilisation of replication forks. Cellular resistance to PARPi is usually caused by the coexistence of several of these mechanisms (54).

Reverse mutations, i.e., those that restore BRCA gene functionality, restore the open reading frame (ORF) previously shifted by the mutation. Long-term exposure to PARP inhibitors can result in the conversion of the mutated BRCA gene to a partially functional gene, resulting in resistance to treatment. The appearance of new mutations can also restore the ORF caused by the previous mutation (34, 55).

Properly functioning BRCA genes are also responsible for stabilizing replication forks. If repair processes stabilize them in ways other than BRCA genes, this may contribute to the progression of resistance. This is because cells with a mutated gene are sensitive to PARPi due to malfunctioning processes. Reducing replication stress and the subsequent stabilisation of replication forks will cause their secondary stabilisation (53, 56).

Exploring the mechanisms of PARP inhibitor resistance is an important task. Understanding these mechanisms allows for the use of combination therapies that will prevent drug resistance in predisposed patients. Many clinical trials are investigating multidrug therapies to reduce the development of resistance (35, 57). Furthermore, the possibility of this complication of therapy has led to research into other drugs that could replace PARPi. One such drug currently being researched is melphalan. This drug has a different mechanism of action than PARP inhibitors. Additionally, like PARPi, it is more beneficial in patients with a mutated BRCA gene than in those with a normal gene (58).

#### 3.6.2 Directions for further scientific research

The development of novel and more effective therapeutic strategies remains a major focus of contemporary oncology research. In addition to investigating agents with mechanisms of action distinct from PARP inhibitors (PARPi), combination therapies are also being extensively studied. Promising results have been reported for combinations of olaparib with anti-PD-L1 agents, as well as olaparib with bevacizumab, both of which demonstrated favorable clinical responses in selected patient groups. However, further large-scale studies are required to confirm the efficacy and safety of these therapeutic approaches (59, 60).

Another important area of research concerns the role of PARPi in maintenance therapy. Although their efficacy in first-line treatment has been well established, prolonged use in second- and third-line settings may contribute to the development of treatment resistance. Consequently, there is a growing need to identify predictive biomarkers that could enable more accurate assessment of treatment response and improve patient selection for PARPi therapy (61). One promising biomarker currently under investigation is HRDsig, which may facilitate evaluation of therapeutic efficacy and help minimize treatment-related toxicity and adverse effects, thereby improving patients’ quality of life (33).

Circulating free tumor DNA (cfDNA) also represents a promising biomarker for the future management of ovarian cancer. Analysis of cfDNA using next-generation sequencing (NGS) enables the detection of BRCA reversion mutations associated with resistance to PARPi (61). Similarly, liquid biopsy techniques provide a non-invasive method for assessing circulating tumor-derived genetic material and may help predict tumor sensitivity to therapy. The integration of cfDNA analysis and advanced liquid biopsy approaches into clinical practice may substantially improve treatment monitoring and therapeutic outcomes in ovarian cancer patients (62, 63).

The broader implementation of BRCA mutation and HRD testing in routine clinical practice remains essential. Delayed genetic assessment, often resulting from poor patient condition or insufficient biological material, may negatively affect survival outcomes. Increasing physician awareness of modern genetic diagnostic strategies is therefore of considerable importance, particularly because molecular testing remains underutilized in many clinical centers despite its direct impact on treatment selection. Further research is also needed to develop faster, more accessible, and more reliable diagnostic methods that can be widely implemented in everyday clinical practice (64). Beyond biomarker testing, innovative immunotherapeutic strategies and novel antibody-drug conjugates, such as mirvetuximab soravtansine, are actively broadening the range of available therapies and shaping future personalized paradigms for ovarian cancer management (65, 66).

### 3.7 Summary

#### 3.7.1 Discussion

Analysis of BRCA1 and BRCA2 genetic alterations has significant implications for the diagnosis, prognosis, and treatment of ovarian cancer. The available evidence demonstrates considerable geographic and population-based variability in both the distribution of histological subtypes and the prevalence of BRCA mutations. Differences are also observed in the relative frequency of BRCA1 and BRCA2 mutations among individual populations. This heterogeneity may partially contribute to the high clinical burden of ovarian cancer, as the dominant histological subtype and the penetrance of BRCA mutations remain insufficiently characterized in many regions of the world. Improved understanding of these epidemiological patterns could facilitate earlier and more accurate prognostic assessment during the diagnostic process. To address these epidemiological gaps, current international diagnostic guidelines should be updated to mandate universal germline and somatic BRCA1/2 testing for all newly diagnosed epithelial ovarian cancer patients, regardless of family history or geographical region. Current screening protocols are often restricted by cost or regional criteria, which leads to underdiagnosis. We suggest transforming the diagnostic framework by integrating rapid NGS)directly into the initial histopathological assessment. Prognostically, guidelines should transition from a binary classification (BRCA-mutated vs. wild-type) to a dynamic prognostic scoring system that incorporates the specific location of the mutation (e.g., ovarian cancer cluster regions) and the variant type, allowing clinicians to predict long-term survival and recurrence risks with significantly higher precision.

Furthermore, prognostic models must be contextualized differently when evaluating BRCA1 versus BRCA2 mutations in ovarian cancer compared to breast cancer. Although both malignancies share a common genetic etiology, the clinical behavior and prognostic implications of these alterations diverge substantially. In ovarian cancer, BRCA2 mutations are consistently associated with a highly favorable initial prognosis and exceptional sensitivity to platinum-based chemotherapy and PARP inhibitors due to profound homologous recombination deficiency, often outperforming BRCA1 carriers in short-term survival. Conversely, BRCA1 mutations in ovarian cancer, while still responsive to targeted therapies, typically present with a higher baseline tumor grade and a faster onset of recurrence. In the context of breast cancer, the prognostic landscape shifts dramatically; BRCA1 carriers predominantly develop aggressive, triple-negative breast cancers with a poor baseline prognosis, whereas BRCA2 carriers are more likely to develop hormone receptor-positive tumors that carry a distinct, protracted long-term risk of contralateral recurrence. Therefore, future clinical guidelines must avoid a generalized “BRCA-carrier” prognostic framework and instead implement organ- and gene-specific, dynamic stratifications that accurately reflect the unique survival trajectories of BRCA1- and BRCA2-associated malignancies.

A similar challenge concerns the association between specific histological subtypes and BRCA mutations. Although high-grade serous ovarian carcinoma is consistently reported as the subtype most strongly associated with BRCA mutations, findings regarding other histological variants remain inconsistent. This represents an important gap in current knowledge that requires further investigation.

BRCA mutations substantially increase the risk of ovarian cancer and are associated with distinct clinical behavior. Patients carrying BRCA mutations demonstrate longer progression-free survival and a more favorable response to platinum-based chemotherapy compared with patients without these alterations. This enhanced treatment sensitivity is primarily related to impaired DNA repair mechanisms caused by homologous recombination deficiency. In this context, PARP inhibitors have emerged as a particularly important therapeutic strategy in BRCA-mutated ovarian cancer. By inhibiting alternative DNA repair pathways, PARP inhibitors promote the accumulation of DNA damage, ultimately leading to cancer cell apoptosis through the mechanism of synthetic lethality. Their introduction into clinical practice has significantly improved progression-free survival and, in many cases, overall survival.

Although BRCA mutation status does not alter the general treatment sequence, which remains based on cytoreductive surgery and chemotherapy, it strongly influences the selection of targeted therapies. Patients without BRCA mutations derive substantially less benefit from PARP inhibitor treatment. Consequently, the clinical value of genetic testing extends beyond prognostic assessment and plays a central role in personalized treatment selection. Moreover, identification of BRCA mutations has important implications for family members, enabling genetic counseling and participation in surveillance programs. In terms of therapeutic management, current clinical guidelines heavily rely on PARP inhibitors primarily in the maintenance setting after first- or second-line platinum-based chemotherapy. However, based on the synthesized evidence, we propose a pivotal shift in these guidelines toward the upfront integration of PARP inhibitors or combination regimens (e.g., combining PARP inhibitors with anti-angiogenic agents or immune checkpoint inhibitors) as an immediate first-line strategy for high-risk BRCA-mutated patients. Furthermore, standard guidelines should be modified to include mandatory, routine HRD testing alongside BRCA screening. This change would expand the targeted treatment eligibility criteria, ensuring that patients who exhibit a “BRCA-ness” phenotype due to epigenetic silencing or other pathway alterations are not wrongfully excluded from receiving highly effective targeted therapies. However, an important limitation remains the lack of universally accessible screening strategies and rapid diagnostic methods for BRCA mutation detection. The development of reliable biomarkers and widely available screening tools may significantly improve early diagnosis and therapeutic decision-making.

Our review also highlights several limitations in current knowledge and clinical practice. Despite the major therapeutic advances associated with PARP inhibitors, resistance to these agents is becoming an increasingly significant clinical problem. As a result, ongoing research is focused on combination treatment strategies and the development of novel agents with different mechanisms of action. An equally important goal is the identification of predictive biomarkers capable of assessing individual response to PARP inhibitor therapy.

Particular attention has recently been directed toward circulating free tumor DNA (cfDNA), which enables non-invasive monitoring of treatment response and resistance mechanisms. Broader implementation of cfDNA analysis in routine clinical practice could help avoid ineffective therapies and reduce unnecessary treatment-related toxicity. In summary, despite substantial progress in the diagnosis and treatment of BRCA-associated ovarian cancer, further advances in molecular diagnostics, targeted therapies, and predictive biomarkers are still required to improve patient outcomes and reduce the mortality associated with this disease. Ultimately, the overarching objective of this comprehensive review is to advocate for a structural revision of existing clinical guidelines. By shifting from generalized treatment sequences to molecularly driven, decentralized protocols that prioritize universal early screening, mutation-specific prognostic models, and aggressive first-line targeted combinations, the medical community can transition from reactive management to proactive, personalized oncology, thereby significantly reducing the global mortality of BRCA-associated ovarian cancer.

This review has several limitations. Only articles published in English were included, which may have resulted in the exclusion of relevant studies published in other languages. In addition, case reports were excluded because the review focused on studies involving larger patient cohorts, thereby ensuring greater reliability and consistency with current clinical guidelines.

#### 3.7.2 Conclusion

Our review summarizes the current state of knowledge regarding the diagnosis and treatment of BRCA-related ovarian cancer. Although substantial progress has been made in understanding the role of BRCA mutations in ovarian carcinogenesis, several important gaps remain. First, while genetic diagnostics undoubtedly facilitate risk stratification and prognostic assessment, further research is needed to develop faster, more accessible, and more accurate screening methods for mutation detection. In many regions worldwide, BRCA testing has not yet become standard clinical practice, highlighting the need to increase awareness of the importance of BRCA mutation assessment in routine patient management.

Furthermore, continued improvement of targeted therapies, particularly PARP inhibitors, remains essential, as the development of treatment resistance continues to represent a major clinical challenge. Future studies should focus on identifying biomarkers predictive of treatment response in order to optimize therapy selection and minimize treatment-related toxicity. Additional research is also necessary to expand the range of effective therapeutic agents and treatment regimens available for patients with BRCA-related ovarian cancer.

Based on the synthesized evidence, we propose several specific recommendations for clinical practice and future research directions. For clinical practice, international guidelines should be updated to mandate universal germline and somatic BRCA1/2 screening via rapid NGS directly during the initial histopathological assessment for all epithelial ovarian cancer patients. Secondly, clinical management should transition from binary BRCA classification to a dynamic prognostic scoring system that factors in the variant type and specific mutation location to better predict long-term survival and recurrence. Furthermore, we suggest shifting the therapeutic paradigm toward the upfront integration of PARP inhibitors or combination regimens as an immediate first-line strategy for high-risk patients, rather than restricting them to maintenance settings. Additionally, implementing mandatory, routine HRD testing alongside BRCA screening is crucial to expand targeted treatment eligibility for patients exhibiting a “BRCA-ness” phenotype.

For future research directions, it is necessary to conduct further regional and multi-center studies to better characterize the penetrance of BRCA mutations, geographic variability, and their specific association with non-HGSC histological variants. Moreover, future clinical trials should focus heavily on deciphering the molecular mechanisms behind PARP inhibitor resistance and developing novel combination treatment strategies to bypass this clinical limitation. Finally, research efforts must be directed toward the identification of predictive biomarkers, specifically accelerating the implementation of circulating free tumor DNA (cfDNA) for non-invasive monitoring of treatment response and early detection of resistance.
